# THE NATIONAL COLLABORATORY TO ADDRESS ELDER MISTREATMENT: A COLLECTIVE IMPACT APPROACH

**DOI:** 10.1093/geroni/igz038.289

**Published:** 2019-11-08

**Authors:** Rebecca Jackson Stoeckle, Kristin Lees-Haggerty, Kim Dash, Mark Lachs, Laura Mosqueda, Alice Bonner, Carmel Dyer

**Affiliations:** 1 Education Development Center, Boston, Massachusetts, United States; 2 Education Development Center, Waltham, Massachusetts, United States; 3 Weill Cornell, New York, New York, United States; 4 Keck School of Medicine University of Southern California, Los Angeles, California, United States; 5 IHI, Boston, Massachusetts, United States; 6 UT Health, Houston, Texas, United States

## Abstract

The National Collaboratory to Address Elder Mistreatment is a team of experts who have come together to develop, test, and disseminate a care model for addressing elder mistreatment in emergency departments. In this presentation we will demonstrate how a collective impact approach, including a centralized infrastructure, dedicated staff, and structured process allowed the Collaboratory to develop and test the Elder Mistreatment Emergency Department Care Model. The model consists of four core elements: 1) The Elder Mistreatment Emergency Department Assessment Profile, 2) staff training modules, 3) brief screening and response tools adapted from a validated measure, and 4) a roadmap for leveraging community resources to support referral and follow up. We will explore the applicability of this approach for harnessing and integrating multidisciplinary expertise to develop, test, and disseminate innovative solutions to complex health care challenges.

